# Clinical use of PET/CT in thyroid cancer diagnosis and management

**DOI:** 10.2349/biij.2.4.e56

**Published:** 2006-10-01

**Authors:** FX Sundram

**Affiliations:** Nuclear Medicine and PET/CT Centre, Subang Jaya Medical Centre, Subang Jaya, Selangor, Malaysia

**Keywords:** Thyroid cancer, thyroglobulin, radioiodine scan, PET/CT

## Abstract

The incidence of thyroid cancer is low, but when it occurs, it is mainly of the papillary histopathological type. Although PET/CT has a limited role in the diagnosis, it plays a significant role in the overall post-surgery management of a patient with thyroid cancer. This follow-up role is important, especially in patients with elevated serum thyroglobulin, but negative radioiodine whole body scans. There is increasing evidence that PET/CT should be a part of routine care in the Tg positive Radioiodine scan negative patient.

## INTRODUCTION

Thyroid cancer is an uncommon disease, with a yearly incidence of about 40 out of 100,000 in women and 15 out of 100,000 in men. The majority of thyroid cancers are of the differentiated type, mainly papillary, and to a lesser extent follicular cancer. For early diagnosis of thyroid cancer, Ultrasonography and fine-needle aspiration biopsy of thyroid nodules is important. Scanning with Tc-99m pertechnetate, Tc-99m sestamibi or tetrofosmin and Thallium-201 Chloride, may help in some cases [1]. The use of 18 F- fluorodeoxyglucose to assess thyroid nodules is still under consideration [2]. Choi et al [3] found focal thyroid lesion in 4% of PET/CT scans, with maximum SUV of malignant thyroid lesions to be significantly higher than that of benign lesions. The cancer risk of focal thyroid lesions was found to be 39%.

Age, sex, tumour stage, and histopathological grading have prognostic significance. Total thyrodectomy is the treatment of choice for differentiated thyroid cancer, and the procedure includes lymph-node dissection of the central compartment. Radioiodine treatment usually follows thyroidectomy, to ablate benign thyroid remnants and destroy remaining malignant cells. Follow-up with serum thyroglobulin levels, sonography of the thyroid bed, and radioiodine scintigraphy is essential, especially to detect local recurrence or distant metastases [4]. There is good correlation between the Tg levels (in the absence of thyroglobulin autoantibody) and persistence of disease. In most cases, undetectable Tg levels suggest absence of either thyroid tissue or distant metastases [4,5]. An elevated serum Tg suggests disease and is usually associated with an abnormal I-131 WBS, with either local recurrence or distant metastases. However, discordant results between I-131 WBS and serum thyroglobulin have been encountered. In particular, approximately 15 to 20% of patients with high serum Tg have negative I-131 diagnostic WBS [5,6]. These false-negative scans may be due to the low dose of iodine administered (diagnostic dose), presence of tumour deposits too small to be detected by a gamma camera, or loss of iodine concentration as a result of tumour dedifferentiation, with impaired sodium-iodide symporter (NIS) system or impaired TSH receptor stimulation. Several recent studies have shown that fluorine-18-fluorodeoxyglucose (FDG) positron emission tomography (PET) can be used to detect local recurrence and distant metastases of thyroid carcinoma, especially in those patients who present with high serum Tg, but negative I-131 WBS [6-8].

## SERUM THYROGLOBULIN (TG) AND RADIONUCLIDE SCANS IN THE FOLLOW-UP OF DIFFERENTIATED THYROID CANCER (DTC)

The follow-up of patients treated for DTC usually includes clinical monitoring, serum thyroglobulin measurements, radionuclide imaging, and anatomic imaging (ultrasonography and CT scans) when indicated. Both Tg measurements and radionuclide imaging rely on TSH stimulation for higher sensitivity. The serum Tg under TSH stimulation (after stopping thyroid hormone for four to six weeks or after two intramuscular injections of recombinant TSH (rhTSH)) is a good indicator of persistent or recurrent disease [8]. The Tg level is reliable only if Tg antibodies are undetectable and recovery of Tg is in the normal range. If high-risk and low-risk groups are differentiated, the consensus is that for low-risk group in complete remission, ultrasonography of the neck and rhTSH stimulated Tg are sufficient for further follow-up [9].

For high-risk patients, many centres perform radioiodine I-131 scans, with Tg under TSH stimulation. The specificity of I-131 whole body scans (WBS) is high, but the sensitivity is low [10]. Radioiodine therapy necessitated by rising Tg levels has a therapeutic effect, and the post-therapy WBS has a higher sensitivity compared with a low-dose diagnostic scan [11]. In some patients, there is elevation of TSH-stimulated Tg, but the diagnostic I-131 WBS is negative. Explanations for false negative WBS could be iodine contamination, insufficient TSH stimulation, small tumour volume, or iodine negative metastases [12]. FDG PET/CT should be considered if the last two reasons are likely. Other tracers, such as, Technetium-99m sestamibi may be used for WBS, especially for soft tissue/nodal disease [13].

## PET/CT IN THE FOLLOW-UP OF THYROID CANCER

**Figure 1:**
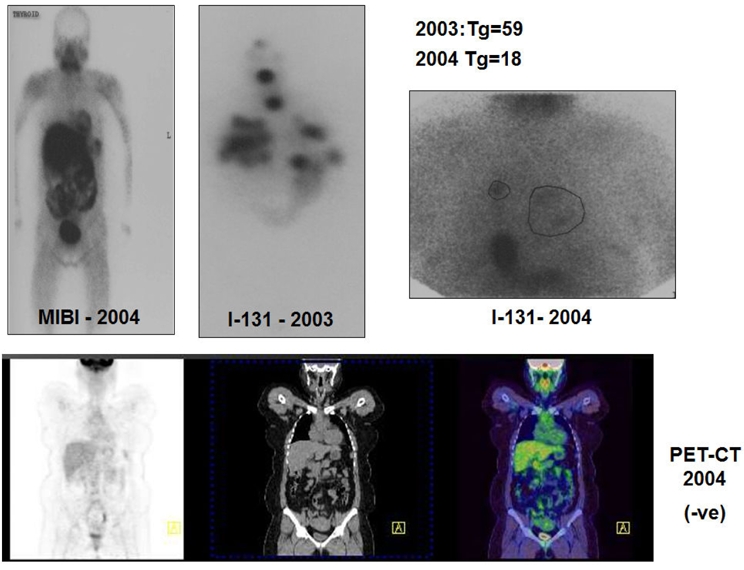
I-131 positive scan with negative PET scan.

**Figure 2:**
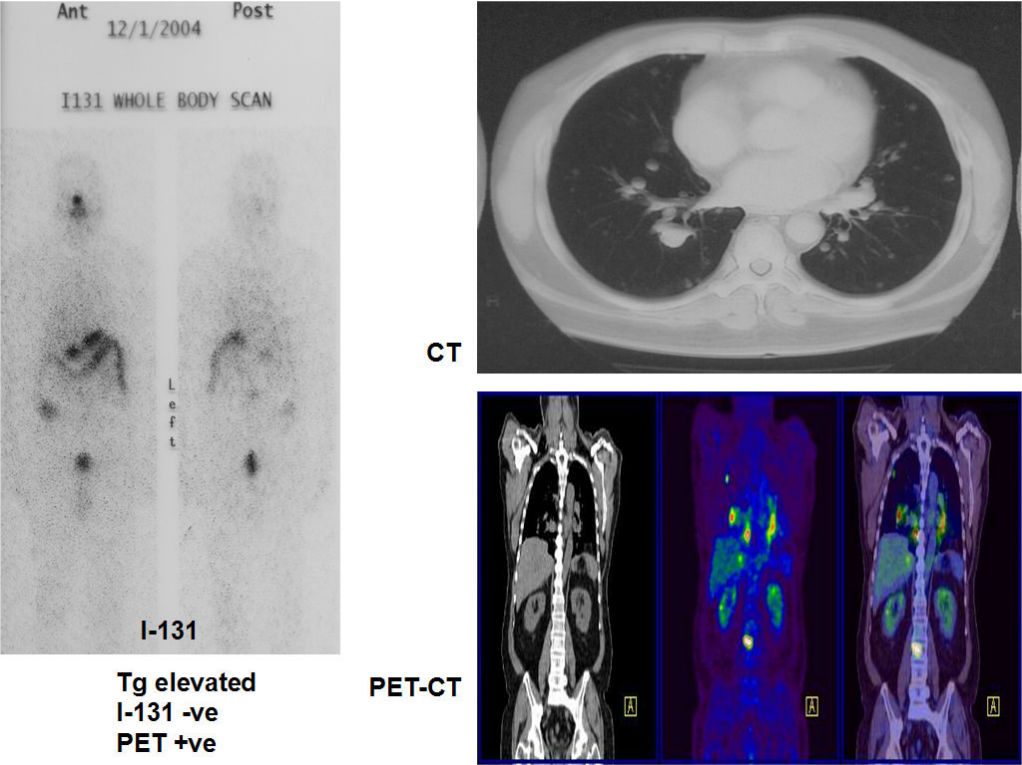
Negative I-131 scan in patient with lung metastasis on CT and PET scans.

Using FDG-PET imaging Feine et al. [14] noted that differentiated tumours with iodine avidity have low glucose metabolism in most patients, with the converse also being true, indicating that high glucose metabolism signifies poor tumour differentiation and higher possible malignant potential. The diagnostic sensitivity of FDG-PET and I-31 WBS combined was found to be 95%. I-131 negative, but FDG-positive or I-131 positive and FDG negative scan was found in 90% of the patients. Examples of this ‘flip-flop’ phenomenon are shown in Figure 1 and Figure 2.

FDG-PET has been advocated as a monitoring procedure for patients with:

1. High-risk disease
2. Adverse histology (e.g., columnar cell, tall cell, and insular variants)
3. Rising Tg levels with no known anatomic source
4. Hurthle cell carcinoma

FDG-PET has also been recommended for post-treatment response assessment, lesion dosimetry, and evaluation of the thyroid nodule, but not recommended for determining extent of disease in low-risk cases.

FDG-PET scans are most useful in ‘high-risk’ patients, wherein tumours are more biologically aggressive, and for metastatic disease. Apart from aiding in diagnosis, PET measurement of glucose metabolism provides biologic information, as noted in the standardised uptake value (SUV). Patients whose cancers take up FDG well are not likely to respond to radioactive iodine. Furthermore, the PET-FDG SUV is a strong predictor of adverse prognosis, with higher SUV’s indicating worse overall prognosis [15].

The usefulness of FDG-PET may depend on factors, such as, Tg level, TSH stimulation by thyroid hormone withdrawal, and TSH stimulation by rhTSH administration. In a study by Schluter et al [16], positive FDG-PET scan results were achieved in 11% of patients with Tg levels of 10ng/ml or less; this increased to 50% among patients with Tg levels between 10 to 20 ng/ml and to 93% at Tg levels above 100ng/ml.

Patients should generally stop thyroid hormone treatment prior to PET scanning. TSH stimulation could be either via hormone withdrawal or by injection of rhTSH. In a study by Petrich et al [17], the sensitivity of FGD-PET was 53% during TSH suppression and 87% following rhTSH stimulation. Their conclusion was that rhTSH FDG-PET suggested specific therapeutic interventions in 57% of patients, with surgery indicated in 23%.

With the introduction of PET/CT imaging for cancer, questions arise as to whether PET/CT imaging is superior to PET and CT alone, and does PET/CT improve patient management compared with both modalities alone. PET can be considered functional (metabolism), while CT mainly reflects anatomy. In combined PET/CT the CT data are used for attenuation correction and anatomic localisation. There is no consensus yet on whether the CT should be done as contrast-enhanced diagnostic CT or as low-dose CT for anatomic correlation. Generally the PET/CT scans are done without contrast, as the SUV values are altered by contrast, which also reduces I-131 uptake when the treatment dose is given. Early clinical results of PET/CT fusion imaging indicate that the exact localisation of hypermetabolic lesions leads to better reliability of results and higher diagnostic confidence, compared with each imaging modality alone [18,19]. In general, for staging and restaging of various tumours, there is additional information in about 45% of patients and change in management in 15% of patients using PET/CT fusion imaging [20]. Furthermore, pitfalls of PET, such as, normal structures in head and neck region, bowel activity, muscle activity, renal excretion, ureteric activity, and brown fat tissue could be reduced by using PET/CT [20].

In our study of thyroid cancer patients with elevated Tg, but negative I-131 WBS, lesions were found in 15 out of 17 patients consistent with metastases, resulting in 88% sensitivity [21]. Nahas et al [22] report a PET/CT sensitivity of 66% in identifying recurrent disease, with a specificity of 100% and a positive predictive value of 100%. These authors conclude that PET/CT is most useful in the detection and the management of recurrent papillary thyroid carcinoma in patients with average Tg levels greater than 10ng/ml. A more recent study by Palmedo et al [23] using PET/CT in patients with suspected iodine-negative differentiated thyroid carcinoma, showed diagnostic accuracy of 93% for PET/CT and 78% for PET alone. They also noted that in tumour positive PET patients, the PET/CT fusion imaging led to a change of therapy in 48% of the patients.

The Centres for Medicare and Medicaid Services (USA) began coverage of FDG-PET procedure in October 2003, for restaging of recurrent or residual thyroid cancer of follicular cell origin that has previously been treated by thyroidectomy and radioiodine ablation in patients with serum Tg levels of 10ng/ml or greater and negative I-131 WBS.

Early this year, the American Thyroid Association produced an excellent set of Management Guidelines for patients with thyroid nodules and differentiated thyroid cancer (24). They recommended that if an empiric dose (100 to 200 mCi) of radioiodine fails to localise persistent disease, FDG-PET should be considered, especially in patients with unstimulated serum thyroglobulin levels more than 10 to 20 ng/ml.

## IODINE-124 PET/CT

Iodine-124 produced in a clinical cyclotron facility can be used to identify mediastinal micrometastases in thyroid carcinoma [25]. Freudenberg et al [26] reported that Iodine-124 PET/CT imaging is a promising technique to improve treatment planning in thyroid cancer. It appears likely that I-124 PET/CT may have an important role for individualised dosimetry in patients with metastatic thyroid cancer [27]. Although I-124 is available in only a few hospitals worldwide, it is an ideal tracer for dosimetry. The treatment dose with Iodine -131 can be planned based on the dosimetric studies done with PET/CT using I-124, especially because the spatial resolution of the PET camera is better than the gamma-camera images using I-131 (Divgi, personal communication).
